# Characterizing Vocal Repertoires—Hard vs. Soft Classification Approaches

**DOI:** 10.1371/journal.pone.0125785

**Published:** 2015-04-27

**Authors:** Philip Wadewitz, Kurt Hammerschmidt, Demian Battaglia, Annette Witt, Fred Wolf, Julia Fischer

**Affiliations:** 1 Cognitive Ethology Laboratory, German Primate Center, Göttingen, Germany; 2 Theoretical Neurophysics, Max Planck Institute for Dynamics and Self-Organization, Göttingen, Germany; 3 Bernstein Center for Computational Neuroscience, Göttingen, Germany; 4 Theoretical Neurosciences Group, Institute for Systems Neuroscience, Marseille, France; University of Cyprus, CYPRUS

## Abstract

To understand the proximate and ultimate causes that shape acoustic communication in animals, objective characterizations of the vocal repertoire of a given species are critical, as they provide the foundation for comparative analyses among individuals, populations and taxa. Progress in this field has been hampered by a lack of standard in methodology, however. One problem is that researchers may settle on different variables to characterize the calls, which may impact on the classification of calls. More important, there is no agreement how to best characterize the overall structure of the repertoire in terms of the amount of gradation within and between call types. Here, we address these challenges by examining 912 calls recorded from wild chacma baboons (*Papio ursinus*). We extracted 118 acoustic variables from spectrograms, from which we constructed different sets of acoustic features, containing 9, 38, and 118 variables; as well 19 factors derived from principal component analysis. We compared and validated the resulting classifications of k-means and hierarchical clustering. Datasets with a higher number of acoustic features lead to better clustering results than datasets with only a few features. The use of factors in the cluster analysis resulted in an extremely poor resolution of emerging call types. Another important finding is that none of the applied clustering methods gave strong support to a specific cluster solution. Instead, the cluster analysis revealed that within distinct call types, subtypes may exist. Because hard clustering methods are not well suited to capture such gradation within call types, we applied a fuzzy clustering algorithm. We found that this algorithm provides a detailed and quantitative description of the gradation within and between chacma baboon call types. In conclusion, we suggest that fuzzy clustering should be used in future studies to analyze the graded structure of vocal repertoires. Moreover, the use of factor analyses to reduce the number of acoustic variables should be discouraged.

## Introduction

Objective classifications of animal signals are a prerequisite for addressing a broad array of questions, both at the proximate and ultimate level. Much progress has been made in developing quantitative methods to objectively characterize single acoustic patterns [1,2]. Less agreement, however, exists on how to objectively characterize the structure of the entirety of a species, that is, its vocal repertoire. Being able to compare the vocal repertoires of different species is crucial to test hypotheses regarding the selective pressures that shape signal repertoires. For instance, the habitat a species lives in was suggested to influence both the spectral characteristics as well as the overall structure of a repertoire [3–5]. More recently, it was suggested that increased social complexity gives rise to increased vocal complexity [6,7]. To rigorously test this assumption, quantitative assessments of vocal complexity are needed. More important, broader comparative or meta-analyses are hampered because studies from different labs often lack consistency in the methods used and in the categorization criteria applied.

Many vocal repertoires are characterized by their graded morphology, meaning that the acoustic structures of vocalizations are not well separated and discrete, but rather form a continuum in the acoustic space [8]. Such graded systems are assumed to have evolved in species with ready visual access to each other [9] and are common in most mammalian vocal systems. Although graded vocal systems are described in a number of nonhuman primates [10–18], labelling whole repertories as being either discrete or graded often represents an oversimplification, since gradation can occur within and between call types, and call types may vary to different degrees [19]. Whereas between-call-type variation might be dependent on the call’s function, within-call-type variation could be linked to an animal’s general affective state [20,21]. Within this general affective state, similar situations can potentially evoke slightly different forms of excitement or fear, which can then relate to dissimilar acoustic structures within call types [22]. The importance to differentiate between these different forms of gradation, however, is neglected in most studies on vocal repertoires.

Whereas historically, vocal repertoires were established by human observers via visual categorization of spectrograms [14], current approaches largely make use of unsupervised clustering methods [10] that are based on acoustic features extracted from spectrograms. The selection and number of these features may have a potentially critical impact on the subsequent analysis. Thus, the question arises whether a quantitative comparison of repertoires is feasible if repertoires are based on different types and numbers of extracted features. In addition, many studies use factors derived from factor analysis to avoid the use of highly correlating acoustic features [18,23,24]. In this study, we use a defined dataset of chacma baboon (*Papio ursinus*) vocalizations to examine how the choice of extracted acoustic features affects clustering results. The structure and function of chacma baboon calls are well known [22,25–27], and were partly validated in playback experiments [28–30]. These previous descriptions of call types allowed us to externally validate the structure of the chacma baboon’s vocal repertoire.

A second focus of this study was to assess how suited different clustering algorithms are to describe the fine structure of graded vocal systems. In a recent study, Kershenbaum and colleagues tested the performance of different unsupervised clustering-algorithms (k-means, hierarchical clustering, and an adaptive resonance theory neural network) for grouping dolphin signature whistles and compared the results with those of human observers [31]. Although all algorithms performed relatively well in the classification of signature whistles, there are some inherent shortcomings that all of them share when constructing vocal repertoires—none of these hard algorithms are able to capture the graded transition of call types that occur in many vocal repertoires. We compared two commonly used non-overlapping models, center-based k-means and hierarchical Ward’s clustering, and opposed them to a soft clustering approach, fuzzy c-means clustering [32]. Fuzzy set theory has a broad range of applications and has for instance been used in numerical taxonomy [33] or to cluster ecological data [34]. Despite its successful application in these fields, it has not yet been used in vocalization taxonomy. Whereas in k-means and Ward’s the existence of a graded separation between call types is not implemented, fuzzy c-means is an algorithm designed to describe systems with not strictly separated categories. We thus expected that fuzzy c-means would be able to describe the graded structure of the chacma baboon’s vocal repertoire better than the other methods.

Our overarching goal is to develop recommendations for future analyses of vocal repertoires, with the long-term perspective of creating unified and standardized procedures in the field of bioacoustic research.

## Methods

### Study site and subjects

In this study, we reanalyzed call recordings that were collected during January 1998 and June 1999 in the Moremi Wildlife Reserve in Botswana. A number of comprehensive studies on the social behavior as well as on the vocal communication of this population has been published (see references in [35]).

### Recordings and call parameterization

Recordings were taken as part of a number of studies on the monkeys’ vocal communication [36]. Vocalizations were recorded with a Sony WM TCD-100 DAT recorder and a Sennheiser directional microphone (K6 power module and ME66 recording head with MZW66 pro windscreen) [36]. We assembled a data set comprising of 912 calls, which we selected to capture the overall diversity of the chacma baboon’s vocalizations. The selected calls were given by 35 adult females and 34 adult males, as well as 5 infant females and 4 infant males (weaning calls). We fast Fourier transformed (FFT) the calls into their frequency-time domain with Avisoft (Avisoft SASLab Pro, version 5.2.05), using a FFT size of 1024 points, Hamming window and 96.87% overlap. Depending on the frequency range of calls we used a sampling frequency of 5 kHz (grunts) or 20 kHz (all others), resulting in a frequency range of 2.5 or 10 kHz and a frequency resolution of 5 or 20 Hz. The time increment was 6.4 or 1.6 milliseconds. The resulting frequency-time spectra were analyzed with the software LMA 2012 developed by Kurt Hammerschmidt.

To assess the influence of datasets with varying numbers of acoustic features on the clustering results, we constructed 4 different sets for the subsequent analyses, all based on the 912 calls in the analysis. The sets include

- “sparse set”: 9 features, which were used in a previous analysis of the Guinea baboon’ vocal repertoire and had proven to be instructive [25]
- “medium set” 38 features, which are an extension of *a)* including more detailed features in the frequency- and time domain
- “full set”: 118 features—the maximum amount of features that can be extracted out of the FFT using LMA
- “factors”: 19 features—derived from a factor analysis of the 118 features dataset.

We performed Factor analysis with IBM SPSS Statistics (version 21) using varimax rotation and factors with an Eigenvalue ≥ 1 were selected. Factor loadings, Eigenvalues, and detailed information about all acoustic features used are given in S1, S2, S3 and S4 Tables.

### Clustering schemes

To classify the calls, we performed unsupervised clustering using the above mentioned feature sets. Sets were standardized by z-scoring all of the values and cluster analysis was run within the Matlab environment (Mathworks; version R2011b). We used different clustering methods for comparison, which are described in the following sections in more detail. First, hard algorithms (k-means, Ward’s clustering) were used and validated. Second, a soft classification scheme based on fuzzy set theory [37] was applied to capture more details of the dataset’s underlying structure.

#### Hard classification models and clustering validation

Ward’s clustering [38] is a hierarchical clustering procedure, that is often used to cluster calls and to analyze vocal repertoires [31,39–41]. The algorithm works by first linking individual calls to their nearest neighbor and then merging the pair of clusters with the minimum between-cluster distance at each time step. This linkage procedure is repeated on these clusters until the top hierarchic level is reached (single-linkage clustering).

In k-means clustering [42], initial cluster centroids are selected randomly and individual calls are assigned to the cluster whose mean yields the least within-cluster sum of squares (WCSS). In iterative steps the new centroids of the clusters are being calculated and the procedure is repeated until the WCSS cannot longer be improved. Since poor initial cluster centroids can lead to non-optimal solutions by running into local maxima, we executed 100 replications to ensure that the best cluster solution was revealed. K-means clustering has the advantage that initially poorly attributed calls are reassigned by the algorithm and is therefore an often used procedure to classify calls [25,31,43,44]. However, since in several studies the determination of the optimal number of clusters k showed to be challenging, we here did a further validation of clustering quality.

To assess which of the feature sets give rise to classifications most robust against changes of the clustering method, we measured the Normalized Mutual Information [32] between clusters extracted by two different methods. Normalized mutual information (NMI) is a single metric that measures how well the results of the two different clustering approaches match. If the clusters extracted by Ward and k-means methods are perfectly overlapping, NMI takes a value of 1. If the resulting clusters have little conformity, NMI takes a positive value close to zero. NMI is defined as:

$$NMI=\frac{\sum_{k,c}n_{k,c}\log\left[\frac{N*n_{k,c}}{n_{k}*n_{c}}\right]}{\sqrt{\left(\sum_{k}n_{k}\log\frac{n_{k}}{N}\right)\left(\sum_{c}n_{c}\log\frac{n_{c}}{N}\right)}}$$

where *n*_*c*_ is the number of calls assigned to cluster c by method 1, *n*_*k*_ is the number of calls assigned to cluster k by method 2, *n*_*k*, *c*_ is the number of calls in cluster c and cluster k, and N is the total number of calls.

**Fig 1:**
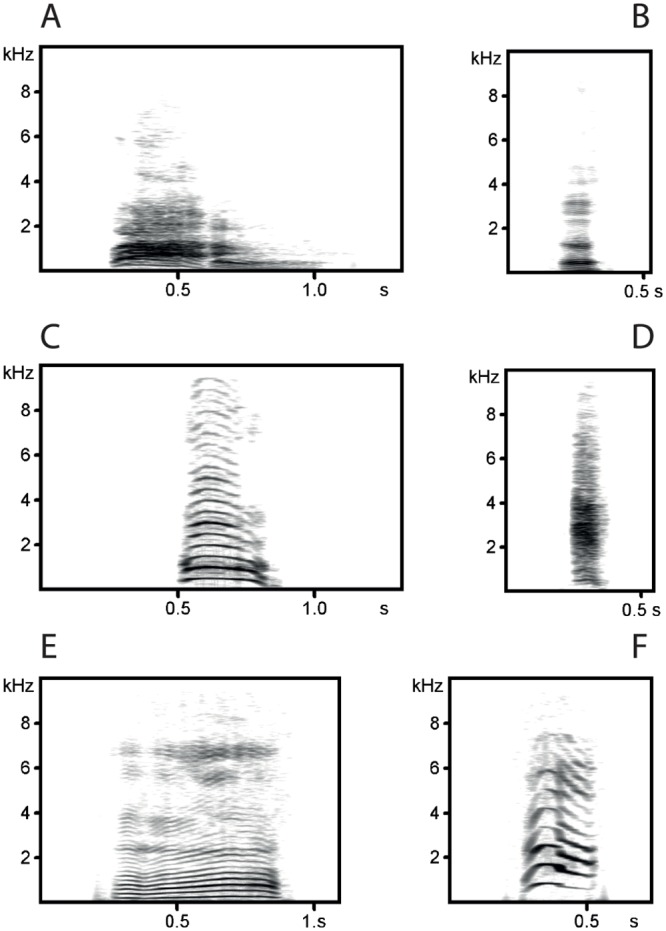
Spectrograms of calls in the used dataset. Shown are call types that have been described in the literature. (A) Male bark [26]. (B) Grunt [27]. (C) Female bark [22]. (D) Noisy scream [25]. (E) Weaning call [25]. (F) Tonal scream [25].

We also used NMI to compare clustering results with a reference classification. Based on prior studies of the usage, function and meaning of vocalizations, we established six call types, namely male barks [26]; grunts [27]; weaning calls [25]; female barks [22]; noisy screams [25]; and tonal screams [25]. Representative calls are shown in Fig 1. Based on acoustic and visual spectrogram evaluation, we assigned each call in the dataset to one of these categories. This procedure provided a defined human expert reference classification.

The quality of a clustering was also be validated by the analysis of silhouette values. Silhouette values range from 1 to -1 and represent the tightness of data points within a cluster and the separation between different clusters in a given model [45]. Silhouette values are computed as following:

$$S(i)=\frac{b(i)-a(i)}{\max[a(i),b(i)]}$$

where *a*(*i*) denotes the average Euclidean distance between data point *i* and other data points in the cluster A and b(*i*) denotes the average Euclidian distance between i and points in the second closest cluster. A silhouette value around zero means that the data point is at similar distance to two clusters. Positive values show that the data point lies closer to one cluster than to the second closest one. Negative values indicate a potential misclassification (even if reassigning a point with a negative silhouette to a different cluster would change as well the cluster means, resulting in a potentially larger number of negative silhouette scores). The overall silhouette width S(A) is defined as the average of the S(i) over the whole dataset and is used as a global measure of the quality of a clustering.

#### Soft classification model: Fuzzy c-means clustering

Fuzzy set theory [37] extends conventional set theory allowing for the notion of imperfect membership. In this way, it is particularly suited to the classification of data in which the separations between different classes of data-points is gradual rather than sharp [46]. Each call is associated an assigned membership value for each of the clusters, ranging from m = 1 (fully displays the properties of the cluster) and m = 0 (does not display any of the properties of the cluster). Intermediate membership values 0 < m_ia_ < 1 mark calls that do not fully belong to one of the clusters, but can be classified as intermediates between different call types. Membership vectors are normalized in such a way that $\sum_{\alpha =1}^{c}m_{i\alpha }=1$.

More specifically, we adopted a fuzzy c-means algorithm [47,48]. To determine the number of clusters that describe the dataset best, two parameters of the algorithm can be adjusted. The first parameter is the maximal number of clusters allowed and the second is the fuzziness parameter μ. If μ = 1, the extracted clusters are very crisp and membership values of data points are either 1 or 0 (in this limit indeed fuzzy c-means converges exactly to k-means). However, by increasing μ, clusters become fuzzier and nearby clusters can eventually merge, unlike in k-means, leading to a smaller number of clusters. We assumed a relatively large possible number of clusters c = 15 (larger than the number of reasonably detectable clusters).

Similar to k-means, the fuzzy c-means algorithm builds up clusters by creating randomly selected cluster centroids and a subsequent iterative optimization process. In this aspect both clustering algorithms suffer from the same sensitivity to the initial cluster centroids. Like in k-means, we computed 100 replications to find the optimal cluster solution with fuzzy c-means. In contrast to k-means, where objects do either belong or not belong to a cluster, in fuzzy c-means membership vectors $m_{i}^{\left(t\right)}$ for c clusters are computed at a given iteration t. Cluster centroids are given by vectors $u_{\alpha }^{\left(t+1\right)}$ (α = 1…c) with components $u_{\alpha l}^{\left(t\right)}$.

$$\frac{1}{m_{i\alpha }^{\left(t\right)}}={\sum_{\lambda =1}^{c}\left(\frac{d_{i\alpha }^{\left(t\right)}}{d_{i\lambda }^{\left(t\right)}}\right)}^{\frac{2}{\mu -1}}$$

where $d_{i\lambda }^{\left(t\right)}$ is the Euclidean distance between the data-point f_i_ and the centroid $u_{\lambda }^{\left(t\right)}$ at a given iteration t.

These membership vectors are used in turn to compute a new set of cluster centroids *u*(*t*+1) a with coordinates:

$$u_{\alpha l}^{\left(t+1\right)}=\frac{\sum_{i=1}^{N}{\left(m_{i\alpha }^{\left(t\right)}\right)}^{\mu }f_{il}}{\sum_{i=1}^{N}{\left(m_{i\alpha }^{\left(t\right)}\right)}^{\mu }}$$

This procedure is designed to minimize a specific cost function [32], namely the sum of the squared distances of the data-points from the different centroids, weighted by the relative fuzzy memberships:

$$J^{t}=\sum_{i=1}^{N}\sum_{\lambda =1}^{c}{\left(m_{i\lambda }^{\left(t\right)}\right)}^{\mu }*{\left(d_{i\lambda }^{\left(t\right)}\right)}^{2}$$

Once the fuzziness parameter μ is set and the clusters (i.e. call types) have been computed, the main type α for each call i is the call type with the highest assigned membership component $m_{i\alpha }=m_{i}{}^{\left(1st\right)}$. By subtracting the second largest membership component $m_{i}{}^{\left(2nd\right)}$ from the first, we get the typicality coefficient $d(i)=m_{i}{}^{\left(1st\right)}-m_{i}{}^{\left(2nd\right)}$ for each call. The average $\bar{d}$ of all typicality coefficients and their distribution, quantified by the halved mean absolute deviation Δ = *d* - *d* / 2 were quantified over the entire dataset. Based on the observed distribution of typicality coefficients, calls were then considered as typical if $d>d_{typical}=\bar{d}+\Delta$ and as atypical if $d<d_{atypical}=\bar{d}-\Delta$.

## Results

**Fig 2:**
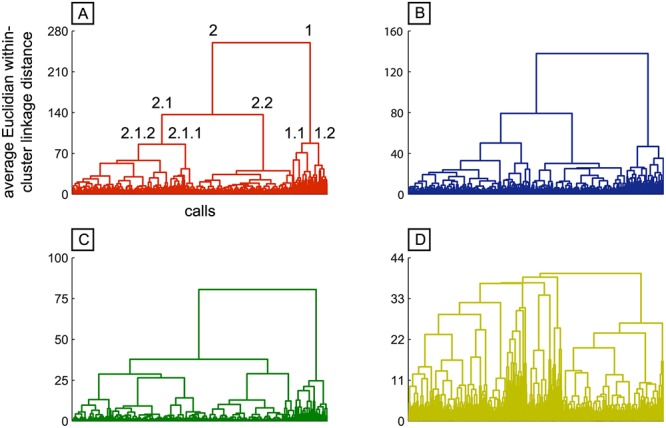
Unsupervised Ward’s clustering of 912 chacma baboon calls based on different frequency dependent and temporal feature setups. The x-axis represents groups of calls, and the y-axis represents average Euclidian within-cluster linkage distance. (A) Set consisting of 118 features. High-frequency (cluster 1) and low-frequency (cluster 2) were segregated into two first-order clusters. High frequency calls further subdivide into more tonal (cluster 1.1) and relatively noisier (cluster 1.2) calls. Low frequency calls subdivide into short and very low-frequency grunt-calls (cluster 2.2), moderate-frequency and harmonic weaning-calls (cluster 2.1.1), and more noisy, short bark-calls (cluster 2.1.2). (B) Set consisting of 38 features. (C) Set consisting of 9 features. (D) Set consisting of 19 factors determined by factor analysis.

The hierarchical clustering trees generated by Ward’s method show similar classifications of calls for all four sets (Fig 2). However, crucial differences in linkage distances can be found (see y-axes of the four graphs). In the following, the results are exemplified for the full set. Calls are first segregated into two clusters. All calls of cluster 1 (n = 124) are characterized by their high frequency distribution over the entire call and are hereafter denoted as “screams”. In contrast, all calls of cluster 2 (n = 788) are characterized by a substantially lower overall frequency distribution. Cluster 2 was further divided into two second-order branches. Cluster 2.2 (n = 350), is characterized by very short and low-frequency calls (grunts). In the next higher order, cluster 2.1 (n = 438), splits into cluster 2.1.1 (n = 97) and cluster 2.1.2 (n = 341). Calls in cluster 2.1.1 are characterized as highly tonal, long and little frequency-modulated (weaning calls), whereas calls in cluster 2.1.2 are shorter and have a higher change in frequency-modulation (barks). On the next level, cluster 1 (screams), is split into cluster 1.1 (n = 68) and cluster 1.2 (n = 56). Calls between these two sub-clusters differ mainly in their signal to noise ratio (STNR), with calls in cluster 1.1 having a higher average STNR. Further structure was detected by the hierarchical clustering. However we did not analyze it in further detail, due to the instability of these classifications (as revealed by fuzzy c-means, see below). Since the Euclidean distance is defined as the square root of the sum of the squared distances per feature, the less features are included in the analysis, the smaller the average Euclidean distance within a cluster becomes (Fig 2). Although the within-cluster distances are decreasing with decreasing number of acoustic features, the separation of the first three clusters remains rather distinct (see branch structure of dendrograms in Fig 2A–2C). An exception of this pattern is formed by the factorial dataset, which shows a much worse separation of even a small number of call clusters (Fig 2D).

**Fig 3:**
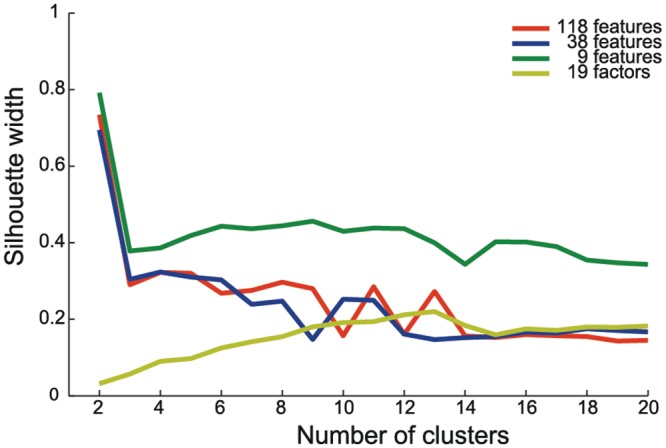
Comparison between the average silhouette width for K-means clustering for k = 2 to 20 clusters for all 4 feature sets. The 9 feature set (green) shows generally higher silhouette width. For the 2-cluster-solution, all but the set based on factors (yellow) show globally the highest value. Excluding the 2-cluster-solution (not to be retained because of its lack of detail), no solution is markedly superior over all others, although plateau values of average silhouette width are already obtained for cluster numbers as small as k = 5 (apart from factor-based clustering).

To compare the clustering quality of the four feature sets, we validated the results of k-means clustering. For this purpose we calculated silhouette widths for k = 2–20 clusters for all four datasets (Fig 3). The general trend for all datasets but the one based on factors was that a 2-cluster solution gained a relatively high value in silhouette widths, followed by a drop and a subsequent stable cluster quality that decreased slowly the more clusters were generated. Silhouette widths for the 9-feature set were generally higher than for the other datasets and silhouette widths for the 19-factor set were generally lower for lower number of cluster solutions.

We then evaluated the large-scale behavior of the four considered curves. Here we found, for the sets with 38 and 118 features slightly decreasing silhouette widths (for more than two clusters), for the set with 9 features essentially constant values (for more than two clusters) and for the set with 19 factors an increase up to 13 clusters that was followed by saturation. For these reasons, Normalized Mutual Information (NMI) was calculated to further explore the quality of clustering results. If our two unsupervised methods (k-means and Ward’s), operating on opposite approaches result in a similar classification, this would be a strong indicator for the robustness of the classification. Classifications extracted by the different methods were overall highly consistent between both algorithms over a wide range of cluster numbers, with peak consistencies for all four datasets nearby k = 5 (excluding, as in Fig 3, the too unresolved k = 2 clustering).

**Fig 4:**
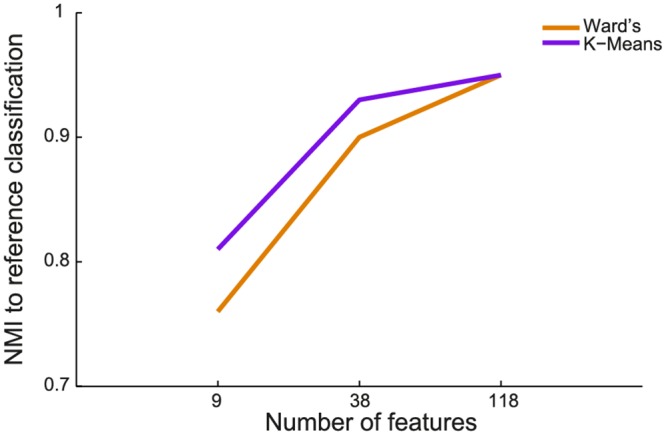
Sensitivity of the algorithm performance (normalized mutual information) between the human-made reference classification and K-means (purple), and Ward’s (orange) clustering for the three feature sets. NMI values have been calculated for k = 5 clusters.

As a final check, since we know from previous studies that the call types of the 5 cluster solution (screams, barks, weaning calls and grunts) are well described calls in baboon vocalizations, NMIs between the 5-cluster partition extracted by k-means or Ward’s unsupervised clustering and the human expert-based reference classification were also calculated (Fig 4). The results show, that the classifications generally match well. This confirms that the 5-cluster solution obtained through k-means and Ward’s methods are consistent with the results obtained by human expert inspection allowed us endorsing the unsupervised methods as valid alternatives to human inspection when the size of the dataset becomes prohibitively large to be manually parsed. The increase of NMI from the 9-feature set to the 38-feature set is quite large for both clustering algorithms, whereas the 118-feature set only gains a small increase in NMI compared to the 38-feature set. Thus, as a compromise between clustering quality and feature overview, we decided to work with the 38-feature set for the subsequent analysis. We decided against a subsequent usage of the 19-factor set, because factors not only showed the worst separation of clusters (Fig 2), but also because factors are difficult to interpret if feature types are highly mixed (see discussion and S4 Table).

### Fuzzy c-means clustering

**Fig 5:**
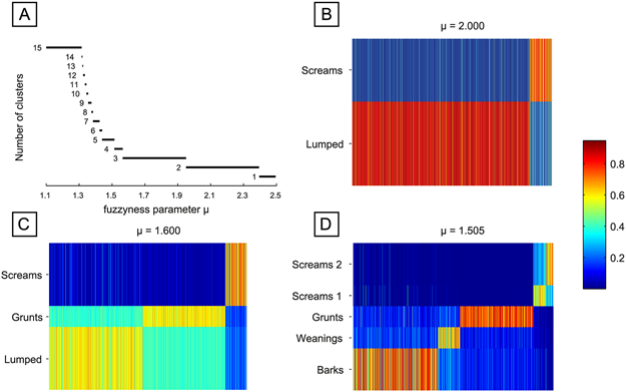
Fuzzy partitions with decreasing fuzziness (μ values) are visualized as membership matrices. (A) Number of clusters in dependence on the fuzziness parameter μ. Partitions with more than five clusters exist only over very narrow ranges of μ values (red). (C-D) membership matrices for the identified clusters: Rows correspond to different fuzzy clusters and columns to individual calls. Membership values of single calls to each class are color coded (B). The scream-cluster is the first to emerge (cluster 1, C), followed by grunts (cluster 2, D). The scream-cluster splits into two clusters and the weaning-cluster emerges (cluster 1–2; cluster 4, E).

To gain better insight into the graded structure of our dataset, we applied fuzzy c-means clustering. This allowed us to determine the best number of clusters in an alternative to silhouette widths for different cluster solutions in the aforementioned algorithms. Hereby, we followed an approach described in [49], where we made use of the fuzziness parameter μ. By starting with a sufficiently large μ, all calls were grouped indistinctively into one fuzzy class. Decreasing the fuzziness, high-frequency calls (“screams”) separated then first at μ = 2.38 (Fig 5A and 5C). At μ = 1.96, a second cluster crystallized, consisting of short and low-frequency calls (“grunts”) (Fig 5A and 5D). Between μ = 1.565 and 1.515, a third cluster of modulated, short and harsher “bark” calls separated and at μ = 1.51, the high-frequency “scream”-cluster split between calls with a higher and lower signal-to-noise ratio (Fig 5A and 5E). Below μ = 1.44 down to μ = 1 several smaller clusters emerged that were not very stable over μ. Looking at stability (cluster existence over fuzziness parameter μ), the 2-, 3- and 5-cluster solutions are most robust (Fig 5A). These results go along with the findings of k-means and Ward’s clustering analyses. In Fig 5C–5E membership values for all calls to the existing clusters are shown for selected values of μ. The remaining analyses were performed for the specific classification obtained for μ = 1.505, leading to 5 clusters. The results were very similar to the results of the k-means and Ward’s clustering, which provides as a strong indicator that the obtained classification is very robust.

**Fig 6:**
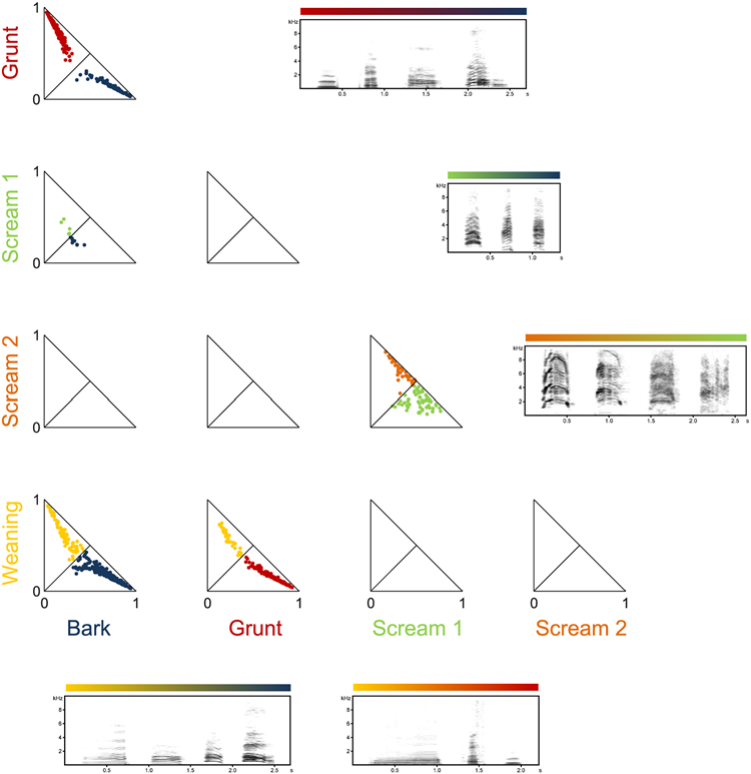
Pairwise comparisons of cluster segregations. Two-dimensional projections of memberships of calls belonging to the grunt (red), scream 1 (green), scream 2 (pink), weaning (yellow), and bark (blue) cluster. Every call is represented once (by closest and second closest cluster). Diagonal lines in the panels represent identical memberships. Spectrograms represent transitions from most typical call of cluster A to most typical call of cluster B with hybrids close to the joint cluster borders. Sound examples can be found in the supporting information.

In Fig 6 a 2-dimensional visualization of how calls are scattered in the membership space is presented. Each call is represented by the closest and the second closest cluster. For the five considered calls types we found common boarders between weaning-calls and barks and weaning-calls and grunts. In both cases, highly typical calls can be found along with calls that appear to belong to both clusters. Intermediate calls can also be found between the two scream types and sparsely between the bark and the scream 1 cluster. Calls in the bark- and grunt-clusters share common boarders and typical grunts and barks exist. In contrast to the other pairs, no calls at the very edge to the other cluster can be found and the two clusters remain separated.

**Fig 7:**
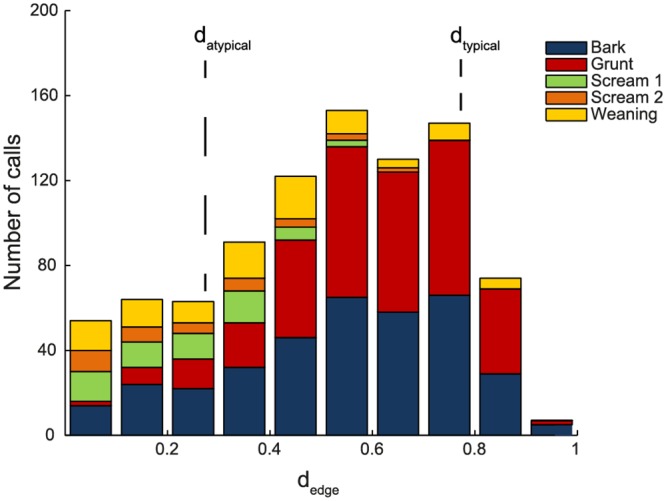
Histogram of typicality coefficients. Sections with different colors indicate calls with different main type. Grunts and barks are more distinctly separated from other call types than screams and weaning calls.

To quantitatively describe the graded structure of our dataset, typicality coefficients for each call were calculated (Fig 7; see Methods). Calls with a typicality larger or smaller than specific thresholds, related to the halved mean absolute deviation of the typicality distribution, were considered as typical or atypical, respectively. According to these criteria, the threshold for atypical calls was calculated at d_atypical_ = 0.256 (142 of 912 ≙ 16% of the calls) and for typical calls at d_typical_ = 0.767 (120 of 912 ≙ 13% of the calls). However, the distribution of typical and atypical calls was not homogeneous across different clusters. Most grunts and the majority of bark-calls were well-separated from the other call types, as indicated by their large average typicality coefficients (Fig 7). Weaning calls were less detached and the two scream clusters were highly graded towards their shared borders.

## Discussion

We investigated how different feature setups can affect the clustering quality, and compared the usage of hard and soft clustering methods for the description of a primate vocal repertoire and. Our efforts provided two key results. Firstly, datasets with a higher number of acoustic features led to better clustering results than datasets with only a few features. Secondly, in datasets with considerable gradation within and between clusters, an optimal number of clusters (call types) may not exist, no matter which clustering algorithm is applied. Yet, fuzzy clustering allows one to capture and quantify the extent of variation within and between clusters, providing a potentially fruitful avenue to compare the extent of gradation within and between call types between taxa.

With regard to the number and types of features in the analysis, we found that a low number of features resulted in higher silhouette values. This was not necessarily due to a better separation of the call types, but rather the consequence of a smaller number of acoustic dimensions, and therefore a higher statistical spread of values. For this reason, the usage of absolute silhouette values to compare datasets with varying number of features does not appear to be appropriate. Indeed, when we compared the human-expert reference classification with the cluster solution, we found that the matching success increased with an increasing number of acoustic features. We therefore recommend the usage of a sufficiently large set of features to capture the different acoustic dimensions. Whereas correlated features can cause problems in multivariate statistical hypothesis testing due to colinearity, these restrictions do not apply to clustering procedures. In fact, correlating features can perform badly in classifying call types when taken on their own, but become well performing classifiers when combined. Since every feature has independent measurement noise that can hinder its classification performance, two or more features can share correlating trends but not the stochastic fluctuations around these trends [50].

We also found that using factors derived from factor analysis resulted in an extremely poor resolution of emerging call types. In addition to the argument above, that correlating features can provide a sort of ‘error correction’ for measurement noise, the weak performance of the factor analysis can be explained by its linear nature, always being based on a matrix decomposition of the covariance matrix. If the established clusters have non-spherical shapes in high-dimensional feature space it might not be possible to properly separate them by hyperplanes orthogonal to the factors. Thus reducing the dimensionality of the data by projecting them to the linear space spanned by only a few factors may conceal non-linear correlations in the data-set, which on the contrary can be exploited for performing clustering by unsupervised algorithms operating on an even smaller number of the original, not factor-reduced features. For these reasons, we generally discourage the use of factors in cluster analysis, and recommend caution when used in acoustic analyses more generally. Factors can be difficult to interpret, especially when highly divergent feature types load onto the same factors (see S4 Table). In such cases, the usage of selected features, preferably derived from a good understanding of the sound production mechanisms [51], is more advisable. If factors are extracted, we recommend inspecting the factors and factor loadings carefully. If parameters load in an interpretable way onto a few factors that explain most of the variance of the dataset, then working with factors may be feasible, but it may also be the case that the construction of apparently meaningful factors results in the loss of crucial variation that would be helpful to distinguish between calls or call types.

A second important insight is that in datasets with a considerable variation an obvious optimal number of call types may not exist. Although the call types in our analysis were easy to distinguish, neither k-means nor Ward’s clustering were able to identify an obvious ‘best solution’. Based on the silhouette coefficients, different cluster solutions appeared to be appropriate to partition the dataset. In this aspect, fuzzy c-means clustering did not facilitate the decision on the best cluster solution. The finding that none of the applied approaches gave strong support to a specific cluster solution is somewhat surprising, since the chacma baboon vocal repertoire was previously described as representing a rather discrete system and call types can be easily categorized by human experts. With fuzzy c-means clustering, the 5-cluster solution was the most stable solution for k>2, but differences in cluster stability were relatively small. A 5-cluster solution was also supported by high silhouette values in k-means and the NMI for call classification between k-means and Ward’s also had an average peak at the 5-cluster solution. Overall, there appeared to be a trade-off between stability and acuity in our analysis.

When inspecting silhouette values, researchers should be aware that these values are affected by a number of factors. Firstly, with increasing number of features, the dimension of the acoustic space is increased. This leads to higher dispersion within and between clusters and consequentially to smaller silhouette widths. Secondly, although for this reason silhouette widths might be high for low feature sets, these sets may miss some crucial acoustic features to separate between different call types and therefore the clustering does not represent the true structure of the vocal system. Thirdly, within one feature set, silhouette widths indicate which cluster solutions are qualitatively better than others. Nevertheless, if the highest silhouette width commends a low number of clusters, this might be mathematically the best solution, but might not provide sufficient detail to describe a species’ vocal repertoire.

Soft clustering allowed us to capture details of the graded nature of vocal repertoires that hard methods did not. Since fuzzy memberships directly represent structural differences of calls, typical and atypical calls within huge datasets can easily be detected and visualized. We propose that the robustness of cluster solutions over the fuzzy parameter in fuzzy c-means clustering (Fig 5A) should be used in future studies to compare differences in the gradation of vocal repertoires between species on a first level. We further showed that the variation in the level of gradation within and between call types can be visualized and even quantified by calculating typicality scores for each call. Whereas the visualization presents a good overview of the repertoire structure, quantification even allows the systematic comparison of the level of gradation between different species’ repertoires.

In sum, although it would be desirable to have completely objective criteria to determine the optimal number of call types, this may not be possible. Therefore, especially in more graded datasets, the researcher’s preference to use different features, or to either split or lump data [52], may also come into play. Transparency with regard to these decisions and awareness of their consequences is therefore invaluable.

## Summary

We conclude that the usage of a high number of acoustic features results in better cluster solutions. The use of factors derived from PCA may result in the loss of critical information and may lead to extremely poor solutions. We therefore discourage their usage for the construction of vocal repertoires. We also showed that fuzzy clustering is a powerful tool to describe the graded structure of a species vocal repertoire. It reveals details of the graded nature of vocal repertoires that cannot be captured with classical approaches and allows a quantification of typical and atypical calls. Researchers should be aware of and transparent about the fact that the outcome of their analysis is affected by several decisions and that the choice of the eventual cluster solution eventually depends on researcher preferences and research interests. Therefore, data repositories should be used so that the same methods can be applied to different datasets. This would greatly enhance the possibilities to compare species’ vocal repertoires within and across taxa.

## Supporting Information

S1 Fig Call exemplars of typical and hybrid calls. (A) Grunt to bark. (B) Tonal scream to noisy scream. (C) Weaning call to bark. (D) Weaning call to grunt. (E) Bark to noisy scream. Colors represent the color code for call types in Figs 6 and 7.
(TIF)

S1 Sound Grunt to bark. (WAV)

S2 Sound Tonal scream to noisy scream. (WAV)

S3 Sound Weaning call to bark. (WAV)

S4 Sound Weaning call to grunt. (WAV)

S5 Sound Bark to noisy scream. (WAV)

S1 Table Descriptions of all 118 acoustic features that were used in the analyses. (DOCX)

S2 Table Eigenvalues of first 20 factors. Extraction Method: Principal Component Analysis.
(DOCX)

S3 Table Scree Plot Eigenvalues of 118 factors. (DOCX)

S4 Table Rotated Component Matrix. Extraction Method: Principal Component Analysis; Rotation Method: Varimax with Kaiser Normalization; Rotation converged in 21 iterations.
(DOCX)
