# Supplementary figures and images for: Characterizing Vocal Repertoires—Hard vs. Soft Classification Approaches
Source: PLoS One. 2015 Apr 27;10(4):e0125785. doi: 10.1371/journal.pone.0125785 (PMC4411004; doi:10.1371/journal.pone.0125785)

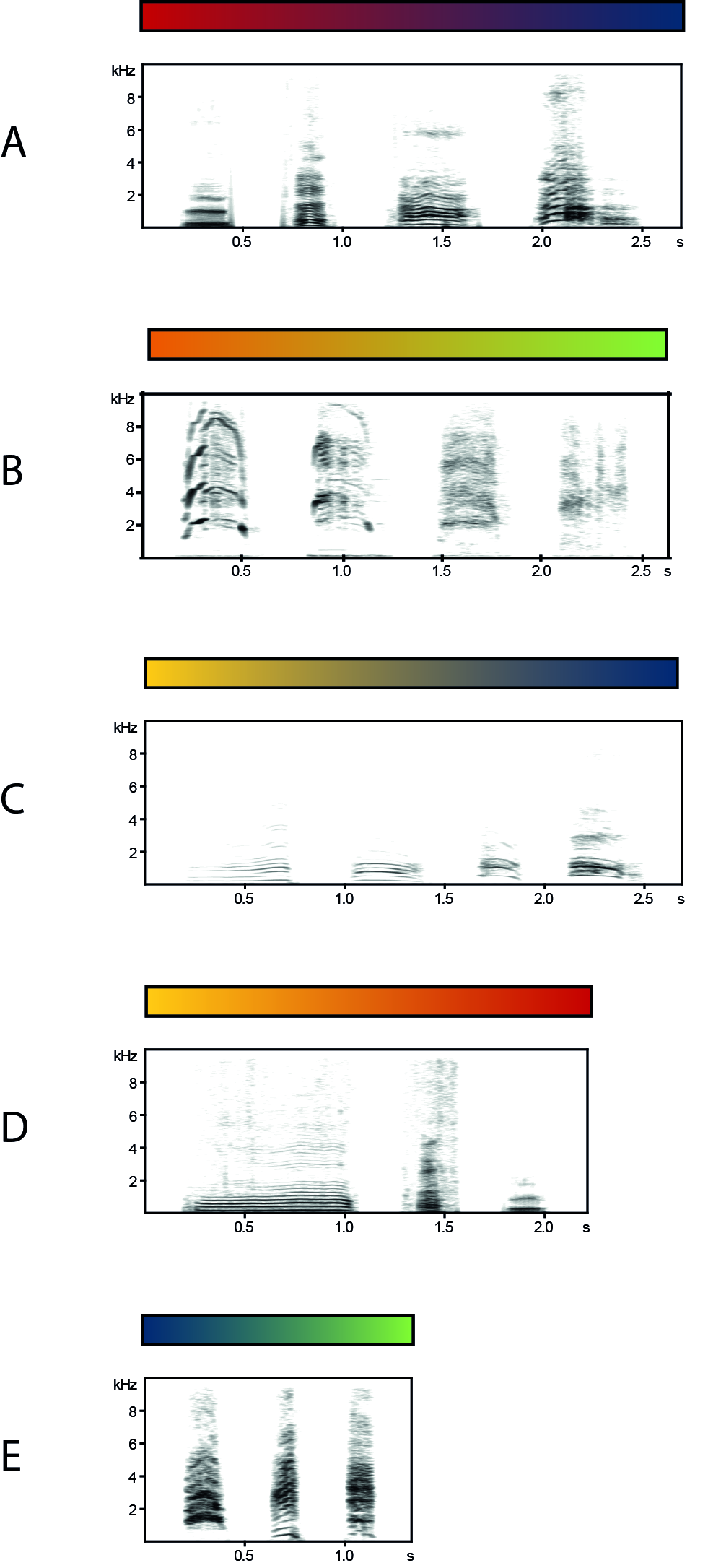

Supplement: S1 Fig — (A) Grunt to bark. (B) Tonal scream to noisy scream. (C) Weaning call to bark. (D) Weaning call to grunt. (E) Bark to noisy scream. Colors represent the color code for call types in Figs 6 and 7. (TIF) [file pone.0125785.s001.tif]

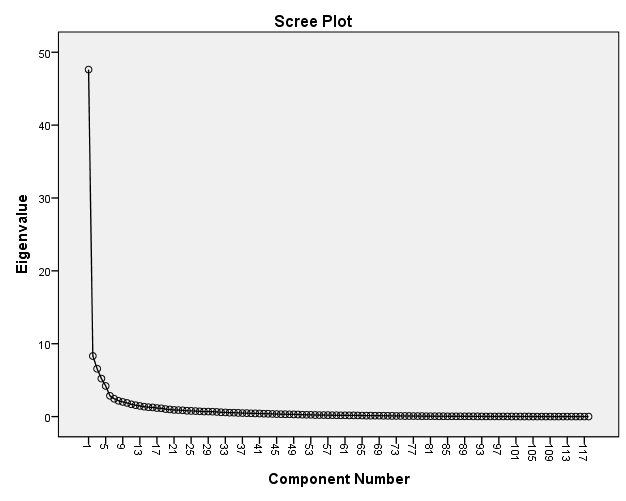

Supplement: S3 Table — (DOCX) [file pone.0125785.s009.docx]
