# Supplementary material for: Characterizing Vocal Repertoires—Hard vs. Soft Classification Approaches
Source: PLoS One. 2015 Apr 27;10(4):e0125785. doi: 10.1371/journal.pone.0125785 (PMC4411004; doi:10.1371/journal.pone.0125785)
Supplement: S1 Table — (DOCX) [file pone.0125785.s007.docx]

| **parameter** | **used in 9- / 38-feature set** | **description and unit** |
| --- | --- | --- |
| Duration | 9 / 38 | Duration [ms] |
| DFA1 st |  | Start frequency 1^st^ DFA (distribution of frequency amplitude) [Hz] |
| DFA1 end |  | End frequency 1^st^ DFA [Hz] |
| DFA1 max |  | Maximum frequency 1^st^ DFA [Hz] |
| DFA1 min |  | Minimum frequency 1^st^ DFA [Hz] |
| DFA1 mean |  | Mean frequency 1^st^ DFA [Hz] |
| DFA1 med |  | Median frequency 1^st^ DFA [Hz] |
| DFA1 maloc |  | Location of the maximum frequency 1^st^ DFA [(1/duration)*location] |
| DFA 2st |  | Start frequency 2nd DFA (distribution of frequency amplitude) [Hz] |
| DFA2 end |  | End frequency 2^nd^ DFA [Hz] |
| DFA2 max |  | Maximum frequency 2^nd^ DFA [Hz] |
| DFA2 min |  | Minimum frequency 2^nd^ DFA [Hz] |
| DFA2 mean | 9 /38 | Mean frequency 2^nd^ DFA [Hz] |
| DFA2 med |  | Median frequency 2^nd^ DFA [Hz] |
| DFA2 maloc | 38 | Location of the maximum frequency 2^nd^ DFA [(1/duration)*location] |
| DFA3 st |  | Start frequency 3rd DFA (distribution of frequency amplitude) [Hz] |
| DFA3 end |  | End frequency 3^rd^ DFA [Hz] |
| DFA3 max |  | Maximum frequency 3^rd^ DFA [Hz] |
| DFA3 min |  | Minimum frequency 3^rd^ DFA [Hz] |
| DFA3 mean |  | Mean frequency 3^rd^ DFA [Hz] |
| DFA3 med |  | Median frequency 3^rd^ DFA [Hz] |
| DFA3 maloc  DFA range | 38 | Location of the maximum frequency 3rd  DFA [(1/duration)*location]  DFA3 mean – DFA1 mean [Hz] |
| DFB1 st |  | start frequency 1^st^ DF (dominant frequency band) [Hz] |
| DFB1 end |  | end frequency 1^st^ DF[Hz] |
| DFB1 max |  | maximum frequency 1^st^ DF [Hz] |
| DFB1 min |  | minimum frequency 1^st^ DF [Hz] |
| DFB1 mean | 9 / 38 | mean frequency 1^st^ DF [Hz] |
| DFB1 med |  | median frequency 1^st^ DF [Hz] |
| DFB1 chfre | 38 | number of changes between original and floating average curve local modulation (LM) 1^st^ DF |
| DFB1 chmea | 9 / 38 | mean deviation LM 1^st^ DF [Hz] |
| DFB1 chmax |  | maximum deviation LM 1^st^ DF [Hz] |
| DFB1 pr | 38 | percent of time segments where a 1^st^ DF could be found [%] |
| DFB1 maloc | 38 | location of the maximum frequency 1^st^ DF [(1/duration)*location] |
| DFB1 miloc | 38 | location of the minimum frequency 1^st^ DF [(1/duration)*location] |
| DFB1 trfak | 38 | factor of linear trend of 1sr DF (global modulation) |
| DFB1 fretr | 38 | alternation frequency between 1^st^ DF and linear trend |
| DFB1 maxtr | 38 | maximum deviation between 1^st^ DF and linear trend [Hz] |
| DFB1 mintr |  | minimum deviation between 1^st^ DF and linear trend [Hz] |
| DFB2 st |  | start frequency 2nd DF (dominant frequency band) [Hz] |
| DFB2 end |  | end frequency 2^nd^ DF [Hz] |
| DFB2 max |  | maximum frequency 2^nd^ DF [Hz] |
| DFB2 mean | 38 | mean frequency 2^nd^ DF [Hz] |
| DFB2 med |  | median frequency 2^nd^ DF [Hz] |
| DFB2 pr |  | percent of time segments where a 2^nd^ DF could be found [%] |
| DFB3 mean | 38 | mean frequency 3^rd^ DF [Hz] |
| DFB3 med |  | median frequency 3^rd^ DF [Hz] |
| DFB3 pr |  | percent of time segments where a 3rd DF could be found [%] |
| DFB4 pr |  | percent of time segments where a 4th DF could be found [%] |
| Diff max |  | maximum difference between 1^st^ & 2^nd^ DF [Hz] |
| Diff mean | 38 | minimum difference between 1^st^ & 2^nd^ DF [Hz] |
| Diff remax |  | maximum number of DF’s |
| Diff remin |  | minimum number of DF’s |
| Diff req | 38 | mean number of DF’s |
| Ampratio 1 |  | amplitude ratio between 1^st^ & 2^nd^ DF |
| Ampratio 2 |  | amplitude ratio between 1^st^ & 3^rd^ DF |
| Ampratio 3 |  | amplitude ratio between 2^nd^ & 3rd DF |
| F1 mean | 38 | (global frequency peak) [Hz] |
| F2 mean | 38 | [Hz] |
| F1 wst |  | start frequency of 1^st^ P [Hz] |
| F1 wend |  | end frequency of 1^st^ P [Hz] |
| F1 wmax |  | maximum frequency of 1^st^ P [Hz] |
| F1 wmin |  | minimum frequency of 1^st^ P [Hz] |
| F1 wmean | 38 | mean frequency of 1^st^ P [Hz] |
| F1 wmed |  | median frequency of 1^st^ P [Hz] |
| FP1 max |  | maximum frequency 1^st^ P (global frequency peak) [Hz] |
| FP1 mean |  | mean frequency 1^st^ P [Hz] |
| FP1 amax |  | maximum amplitude 1^st^ P (global frequency peak) [rel. amplitude] |
| FP1 amean | 38 | mean amplitude 1^st^ P [rel. amplitude] |
| F2 pr | 38 | percent of time segments where a 2^nd^ P could be found [%] |
| F2 wmean | 38 | mean frequency of 2^nd^ P [Hz] |
| F3 pr | 38 | percent of time segments where a 3^rd^ P could be found [%] |
| Range max |  | maximum frequency range [Hz] |
| Range mean | 9 | mean frequency range [Hz] |
| Range min |  | minimum frequency range [Hz] |
| PF st |  | start PF (peak frequency) [Hz] |
| PF end |  | end PF [Hz] |
| PF max |  | maximum PF [Hz] |
| PF min |  | minimum PF [Hz] |
| PF mean | 38 | mean PF [Hz] |
| PF med |  | median PF [Hz] |
| PF totmax |  | frequency of the total maximum amplitude [Hz] |
| PF totmin |  | frequency of the total minimum amplitude [Hz] |
| PF maloc | 38 | location of the maximum PF [(1/duration)*location] |
| PF miloc  PF jump | 38  38 | location of the minimum PF [(1/duration)*location]  maximum difference between successive PF’s [Hz] |
| PF trfak | 38 | factor of linear trend of PF (global modulation) |
| PF trfre | 38 | alternation frequency between PF and linear trend |
| PF trmean | 9 / 38 | mean deviation between PF and linear trend [Hz] |
| PF trmax |  | maximum deviation between PF and linear trend [Hz] |
| CS mean | 9 / 38 | mean correlation coefficient of successive time segments |
| CS maxd |  | standard deviation correlation coefficient of successive time segments |
| CS maloc | 38 | location of maximum correlation coefficient of successive time segments [(1/duration)*location] |
| F0 mean |  | mean frequency F0 [Hz] |
| Noise | 9 / 38 | percentage of noisy time segments [%] |
| Disturb |  | percentage of disturbed time segments [%] |
| Tonal F0 |  | percentage of tonal time segments and it is possible to estimate the F0 [%] |
| PF mean |  | mean PF (peak frequency) [Hz] |
| PF max |  | maximum PF [Hz] |
| PF min |  | minimum PF [Hz] |
| Diff mean |  | mean difference between F0 & PF [Hz] |
| Diff max |  | maximum difference between F0 & PF [Hz] |
| Diff min |  | minimum difference between F0 & PF [Hz] |
| Amprat1 |  | amplitude ration between F0 & 1^st^ harmonic |
| Amprat2 |  | amplitude ration between F0 & 2^nd^ harmonic |
| Amprat3 |  | amplitude ration between 1^st^ & 3^rd^ harmonic |
| HNR1 mean  HNR2 mean  HNR3 mean | 9 / 38 | mean harmonic to noise ratio DFA1 (1= no noise)  mean harmonic to noise ratio DFA2 (1= no noise)  mean harmonic to noise ratio DFA3 (1= no noise) |
| HNR1 max  HNR2 max  HNR3 max  Shimmer mean  Shimmer max  Jitter mean  Jitter max | 38  38 | max harmonic to noise ratio DFA1 (1= no noise)  max harmonic to noise ratio DFA2 (1= no noise)  max harmonic to noise ratio DFA3 (1= no noise)  mean frequency of vocal fold vibration [Hz]  max frequency of vocal fold vibration [Hz]  mean amplitude of vocal fold vibration  max amplitude of vocal fold vibration |
| Range max  Range min |  | maximum frequency range [Hz]  minimum frequency range [Hz] |
|  |  |  |
