# Supplementary material for: Characterizing Vocal Repertoires—Hard vs. Soft Classification Approaches
Source: PLoS One. 2015 Apr 27;10(4):e0125785. doi: 10.1371/journal.pone.0125785 (PMC4411004; doi:10.1371/journal.pone.0125785)
Supplement: S2 Table — Extraction Method: Principal Component Analysis. (DOCX) [file pone.0125785.s008.docx]

| **Total Variance Explained** | | | | | | |
| --- | --- | --- | --- | --- | --- | --- |
| Component | Initial Eigenvalues | | | Rotation Sums of Squared Loadings | | |
|  | Total | % of Variance | Cumulative % | Total | % of Variance | Cumulative % |
| 1 | 47,619 | 40,355 | 40,355 | 45,231 | 38,332 | 38,332 |
| 2 | 8,314 | 7,046 | 47,401 | 7,365 | 6,242 | 44,573 |
| 3 | 6,569 | 5,567 | 52,968 | 3,765 | 3,190 | 47,764 |
| 4 | 5,221 | 4,425 | 57,393 | 3,760 | 3,186 | 50,950 |
| 5 | 4,207 | 3,565 | 60,958 | 3,728 | 3,159 | 54,109 |
| 6 | 2,854 | 2,419 | 63,377 | 3,491 | 2,958 | 57,067 |
| 7 | 2,439 | 2,067 | 65,444 | 3,162 | 2,679 | 59,747 |
| 8 | 2,172 | 1,841 | 67,284 | 2,787 | 2,362 | 62,109 |
| 9 | 2,016 | 1,708 | 68,993 | 2,747 | 2,328 | 64,437 |
| 10 | 1,873 | 1,588 | 70,581 | 2,342 | 1,985 | 66,421 |
| 11 | 1,683 | 1,426 | 72,007 | 2,295 | 1,945 | 68,367 |
| 12 | 1,561 | 1,323 | 73,330 | 2,277 | 1,930 | 70,296 |
| 13 | 1,462 | 1,239 | 74,569 | 2,275 | 1,928 | 72,224 |
| 14 | 1,357 | 1,150 | 75,718 | 2,003 | 1,698 | 73,922 |
| 15 | 1,293 | 1,096 | 76,814 | 1,765 | 1,496 | 75,418 |
| 16 | 1,256 | 1,065 | 77,879 | 1,737 | 1,472 | 76,890 |
| 17 | 1,169 | ,990 | 78,869 | 1,603 | 1,359 | 78,249 |
| 18 | 1,137 | ,964 | 79,833 | 1,547 | 1,311 | 79,560 |
| 19 | 1,031 | ,874 | 80,707 | 1,354 | 1,148 | 80,707 |
| 20 | ,977 | ,828 | 81,535 |  |  |  |
