# Supplementary material for: Characterizing Vocal Repertoires—Hard vs. Soft Classification Approaches
Source: PLoS One. 2015 Apr 27;10(4):e0125785. doi: 10.1371/journal.pone.0125785 (PMC4411004; doi:10.1371/journal.pone.0125785)
Supplement: S4 Table — Extraction Method: Principal Component Analysis; Rotation Method: Varimax with Kaiser Normalization; Rotation converged in 21 iterations. (DOCX) [file pone.0125785.s010.docx]

|  | Component | | | | | | | | | | | | | | | | | | |
| --- | --- | --- | --- | --- | --- | --- | --- | --- | --- | --- | --- | --- | --- | --- | --- | --- | --- | --- | --- |
|  | 1 | 2 | 3 | 4 | 5 | 6 | 7 | 8 | 9 | 10 | 11 | 12 | 13 | 14 | 15 | 16 | 17 | 18 | 19 |
| Duration |  |  |  |  |  |  |  |  |  | ,781 |  |  |  |  |  |  |  |  |  |
| DAF1 1st | ,910 |  |  |  |  |  |  |  |  |  |  |  |  |  |  |  |  |  |  |
| DFA1 end | ,916 |  |  |  |  |  |  |  |  |  |  |  |  |  |  |  |  |  |  |
| DFA1 max | ,966 |  |  |  |  |  |  |  |  |  |  |  |  |  |  |  |  |  |  |
| DFA1 min | ,907 |  |  |  |  |  |  |  |  |  |  |  |  |  |  |  |  |  |  |
| DFA1 mean | ,973 |  |  |  |  |  |  |  |  |  |  |  |  |  |  |  |  |  |  |
| DFA1med | ,968 |  |  |  |  |  |  |  |  |  |  |  |  |  |  |  |  |  |  |
| DFA1 maloc |  |  |  |  |  |  |  |  |  |  |  |  |  | ,471 |  |  |  | ,346 |  |
| DFA2 st | ,853 |  |  |  |  |  |  |  |  |  |  |  |  |  |  |  |  |  |  |
| DFA2 end | ,849 |  |  |  |  |  |  |  |  |  |  |  |  |  |  |  |  |  |  |
| DFA2 max | ,878 |  |  |  |  |  |  |  |  |  |  |  |  |  |  |  |  |  |  |
| DFA2 min | ,931 |  |  |  |  |  |  |  |  |  |  |  |  |  |  |  |  |  |  |
| DFA2 mean | ,979 |  |  |  |  |  |  |  |  |  |  |  |  |  |  |  |  |  |  |
| DFA2 med | ,973 |  |  |  |  |  |  |  |  |  |  |  |  |  |  |  |  |  |  |
| DFA2 maloc |  |  |  |  |  |  |  |  |  |  |  |  |  | ,761 |  |  |  |  |  |
| DFA3 st | ,724 |  |  |  |  |  | ,430 |  |  |  |  |  |  |  |  |  |  |  |  |
| DFA3 end | ,748 |  |  |  |  |  | ,355 |  |  |  |  |  |  |  |  |  |  |  |  |
| DFA3 max | ,731 |  |  |  | -,347 |  | ,389 |  |  |  |  |  |  |  |  |  |  |  |  |
| DFA3 min | ,933 |  |  |  |  |  |  |  |  |  |  |  |  |  |  |  |  |  |  |
| DFA3 mean | ,933 |  |  |  |  |  |  |  |  |  |  |  |  |  |  |  |  |  |  |
| DFA3 med | ,941 |  |  |  |  |  |  |  |  |  |  |  |  |  |  |  |  |  |  |
| DFA3 maloc |  |  |  |  |  |  |  |  |  |  |  |  |  | ,764 |  |  |  |  |  |
| DFB1 st | ,589 |  |  |  |  | ,475 |  |  |  |  |  |  |  |  | -,305 |  |  |  |  |
| DFB1 end | ,651 |  |  |  |  |  |  |  |  |  |  |  |  |  | ,332 |  |  |  |  |
| DFB1 max | ,783 |  |  |  |  | ,550 |  |  |  |  |  |  |  |  |  |  |  |  |  |
| DFB1 min | ,686 |  |  |  |  |  |  |  |  |  |  |  |  |  |  |  |  |  |  |
| DFB1 mean | ,947 |  |  |  |  |  |  |  |  |  |  |  |  |  |  |  |  |  |  |
| DFB1 med | ,928 |  |  |  |  |  |  |  |  |  |  |  |  |  |  |  |  |  |  |
| DFB1 chfre | ,614 | -,402 |  |  |  |  |  |  |  |  |  |  |  |  |  |  |  |  |  |
| DFB1 chmea | ,709 |  |  |  |  | ,413 |  |  |  |  |  |  |  |  |  |  |  |  |  |
| DFB1 chmax | ,513 |  |  |  |  | ,746 |  |  |  |  |  |  |  |  |  |  |  |  |  |
| DFB1 pr |  |  |  |  | ,818 |  |  |  |  |  |  |  |  |  |  |  |  |  |  |
| DFB1 maloc |  |  |  |  |  |  |  |  |  |  |  |  |  |  | ,456 |  |  |  |  |
| DFB1 miloc |  | -,448 |  |  |  |  |  |  |  |  |  |  |  |  | -,382 |  |  |  |  |
| DFB1 trfak |  |  |  |  |  |  |  |  |  |  |  |  |  |  | ,793 |  |  |  |  |
| DFB1 fretr |  |  |  |  |  |  |  |  |  |  |  |  |  |  |  |  |  |  | ,729 |
| DFB1 mtr | ,736 |  |  |  |  | ,415 |  |  |  |  |  |  |  |  |  |  |  |  |  |
| DFB1 maxtr | ,578 |  |  |  |  | ,725 |  |  |  |  |  |  |  |  |  |  |  |  |  |
| DFB2 st | ,642 |  |  |  |  | ,431 |  |  |  |  |  |  |  |  |  |  |  |  |  |
| DFB2 end | ,696 |  |  |  |  | ,356 |  |  |  |  |  |  |  |  |  |  |  |  |  |
| DFB2 max | ,815 |  |  |  |  | ,390 |  |  |  |  |  |  |  |  |  |  |  |  |  |
| DFB2 mean | ,954 |  |  |  |  |  |  |  |  |  |  |  |  |  |  |  |  |  |  |
| DFB2 med | ,944 |  |  |  |  |  |  |  |  |  |  |  |  |  |  |  |  |  |  |
| DFB2 pr |  | ,343 |  |  | ,722 |  |  |  |  |  |  |  |  |  |  |  |  |  |  |
| DFB3 mean | ,942 |  |  |  |  |  |  |  |  |  |  |  |  |  |  |  |  |  |  |
| DFB3 med | ,913 |  |  |  |  |  |  |  |  |  |  |  |  |  |  |  |  |  |  |
| DFB3 pr |  | ,618 |  |  | ,386 |  |  | ,309 |  |  |  |  |  |  |  |  |  |  |  |
| DFB4 pr |  | ,765 |  |  |  |  |  |  |  |  |  |  |  |  |  |  |  |  |  |
| Diff max | ,639 |  |  |  |  | ,534 |  |  |  |  |  |  |  |  |  |  |  |  |  |
| Diff mean | ,807 |  |  |  |  |  |  |  |  |  |  |  |  |  |  |  |  |  |  |
| Diff remax | ,594 | ,650 |  |  |  |  |  |  |  |  |  |  |  |  |  |  |  |  |  |
| Diff remin |  | ,702 |  |  | ,342 |  |  |  |  |  |  |  |  |  |  |  |  |  |  |
| Diff req |  | ,862 |  |  |  |  |  |  |  |  |  |  |  |  |  |  |  |  |  |
| Ampratio1 |  |  |  |  |  |  |  | -,903 |  |  |  |  |  |  |  |  |  |  |  |
| Ampratio2 |  |  |  |  |  |  |  | -,789 |  |  |  |  | -,379 |  |  |  |  |  |  |
| Ampratio3 |  |  |  |  |  |  |  |  |  |  |  |  | -,761 |  |  |  |  |  |  |
| F1 mean | ,950 |  |  |  |  |  |  |  |  |  |  |  |  |  |  |  |  |  |  |
| F2 mean | ,789 |  |  |  |  |  |  |  |  |  |  |  |  |  |  |  |  |  |  |
| F1 wst | ,357 |  |  |  |  |  |  |  | ,490 |  |  |  |  |  |  |  |  |  |  |
| F1 wend | ,401 |  |  |  |  |  |  |  | ,499 |  |  | ,309 |  |  |  |  |  |  |  |
| F1 wmax | ,837 |  |  |  |  |  |  |  | ,361 |  |  |  |  |  |  |  |  |  |  |
| F1 wmin |  | -,339 |  |  | ,406 |  |  |  | ,364 |  |  |  |  |  |  |  |  |  |  |
| F1 wmean | ,672 |  |  |  |  |  |  |  | ,609 |  |  |  |  |  |  |  |  |  |  |
| F1 wmed | ,566 |  |  |  |  |  |  |  | ,639 |  |  |  |  |  |  |  |  |  |  |
| FP1 max | ,925 |  |  |  |  |  |  |  |  |  |  |  |  |  |  |  |  |  |  |
| FP1 mean | ,944 |  |  |  |  |  |  |  |  |  |  |  |  |  |  |  |  |  |  |
| FP1 amax |  | -,695 |  |  |  |  |  |  |  |  |  |  |  |  |  |  |  |  |  |
| FP1 amean |  | -,724 |  |  |  |  |  |  |  |  |  |  |  |  |  |  |  |  |  |
| F2 pr |  | ,710 |  |  |  |  |  |  |  |  |  |  |  |  |  |  |  |  |  |
| F2 wmean | ,777 |  |  |  |  |  |  |  |  |  |  |  |  |  |  |  |  |  |  |
| F3 pr | ,443 | ,427 |  |  |  |  |  |  |  |  |  |  |  |  |  | ,329 |  |  |  |
| Range mean | ,900 |  |  |  |  |  |  |  |  |  |  |  |  |  |  |  |  |  |  |
| Range max | ,756 |  |  |  | -,302 |  |  |  |  |  |  |  |  |  |  |  |  |  |  |
| Range min | ,507 |  |  |  |  |  |  |  | ,407 |  |  |  |  |  |  |  |  |  |  |
| PF st | ,685 |  |  |  |  |  |  |  |  |  |  | ,367 |  |  |  |  |  |  |  |
| PF end | ,601 |  |  |  |  |  |  |  |  |  |  | ,613 |  |  |  |  |  |  |  |
| PF max | ,931 |  |  |  |  |  |  |  |  |  |  |  |  |  |  |  |  |  |  |
| PF min | ,499 |  |  |  |  |  |  |  |  |  |  | ,702 |  |  |  |  |  |  |  |
| PF mean | ,942 |  |  |  |  |  |  |  |  |  |  |  |  |  |  |  |  |  |  |
| PF med | ,911 |  |  |  |  |  |  |  |  |  |  |  |  |  |  |  |  |  |  |
| PF totmax | ,874 |  |  |  |  |  |  |  |  |  |  |  |  |  |  |  |  |  |  |
| PF totmin | ,683 |  |  |  |  |  |  |  |  |  |  | ,452 |  |  |  |  |  |  |  |
| PF maloc |  |  |  |  |  |  | ,428 |  |  |  |  |  |  |  |  |  |  |  |  |
| PF miloc |  |  |  |  |  |  |  |  |  |  |  |  |  |  |  |  |  | ,731 |  |
| PF jump | ,848 |  |  |  |  |  |  |  |  |  |  |  |  |  |  |  |  |  |  |
| PF trfak |  |  |  |  |  |  |  |  |  |  |  |  |  |  |  |  |  | ,636 |  |
| PF trfre |  | ,327 |  |  |  |  |  |  |  | -,506 |  |  |  |  |  |  |  |  |  |
| PF trmean | ,784 |  |  |  |  |  |  |  |  |  |  | -,342 |  |  |  |  |  |  |  |
| PF trmax | ,849 |  |  |  |  |  |  |  |  |  |  |  |  |  |  |  |  |  |  |
| CS mean |  |  |  |  | ,751 |  |  |  |  |  |  |  |  |  |  |  |  |  |  |
| CS maxd |  |  |  |  | -,638 |  | ,343 |  |  |  |  |  |  |  |  |  |  |  |  |
| CS maloc |  |  |  |  |  |  |  |  |  |  |  |  |  | ,455 |  |  |  | -,305 |  |
| F0 mean | ,704 |  | ,317 |  |  |  |  |  |  |  |  |  |  |  |  |  |  |  |  |
| Noise | ,316 |  |  |  |  |  | -,681 |  |  |  |  |  |  |  |  |  |  |  |  |
| Disturb |  |  |  |  |  |  |  |  |  |  |  |  |  |  |  | ,593 |  |  |  |
| Tonal F0 | -,343 |  |  |  |  |  | ,734 |  |  |  |  |  |  |  |  |  |  |  |  |
| PF mean | ,694 |  |  | ,506 |  |  |  |  |  |  |  |  |  |  |  |  |  |  |  |
| PF max | ,591 |  |  | ,522 |  |  |  |  |  |  |  |  |  |  |  |  |  |  |  |
| PF min | ,498 |  |  | ,536 |  |  |  |  |  |  |  | ,333 |  |  |  |  |  |  |  |
| Diff mean | ,515 |  |  | ,766 |  |  |  |  |  |  |  |  |  |  |  |  |  |  |  |
| Diff max | ,485 |  |  | ,680 |  |  |  |  |  |  |  |  |  |  |  |  |  |  |  |
| Diff min | ,311 |  |  | ,770 |  |  |  |  |  |  |  |  |  |  |  |  |  |  |  |
| Amprat1 |  | -,361 |  |  |  |  |  | ,595 |  |  | ,443 |  |  |  |  |  |  |  |  |
| Amprat2 |  |  |  |  |  |  |  | ,544 |  |  |  |  | ,565 |  |  |  |  |  |  |
| Amprat3 |  |  |  |  |  |  |  |  |  |  |  |  | ,865 |  |  |  |  |  |  |
| HNR1 mean |  | -,696 |  |  |  |  |  |  |  |  |  |  |  |  |  |  |  |  |  |
| HNR2 mean | ,329 |  | ,596 |  |  |  |  |  |  |  |  |  |  |  |  |  |  |  |  |
| HNR3 mean |  |  | ,757 |  |  |  |  |  |  |  |  |  |  |  |  |  |  |  |  |
| HNR1 max | -,341 | -,320 | ,511 | ,342 |  |  |  |  |  | ,305 |  |  |  |  |  |  |  |  |  |
| HNR2 max |  |  | ,815 |  |  |  |  |  |  |  |  |  |  |  |  |  |  |  |  |
| HNR3 max |  |  | ,883 |  |  |  |  |  |  |  |  |  |  |  |  |  |  |  |  |
| Shimmer mean |  | -,321 |  |  |  |  |  |  |  |  | ,753 |  |  |  |  |  |  |  |  |
| Shimmer max |  | -,381 |  |  |  |  |  |  |  |  | ,745 |  |  |  |  |  |  |  |  |
| Jitter mean |  |  |  |  |  |  |  |  |  |  |  |  |  |  |  |  | ,840 |  |  |
| Jitter max |  |  |  |  |  |  |  |  |  |  |  |  |  |  |  |  | ,752 |  |  |
| Range mean | ,637 |  | ,301 | ,395 |  |  |  |  |  |  |  |  |  |  |  |  |  |  |  |
| Range max | ,445 |  | ,354 | ,361 |  |  | ,315 |  |  |  |  |  |  |  |  |  |  |  |  |
| Range min | ,493 | ,379 |  | ,437 |  |  |  |  |  |  |  |  |  |  |  | ,343 |  |  |  |
